# Epidemiological trend and age-period-cohort effects on cardiovascular disease mortality and disability-adjusted life years attributable to dietary risks and high body mass index at the regional and country level across China and Pakistan

**DOI:** 10.3389/fnut.2023.1158769

**Published:** 2023-06-06

**Authors:** Wu Yan, Xiuzhen Yan, Sumaira Mubarik, Nawsherwan

**Affiliations:** ^1^Department of Information, Zhongshan Hospital of Xiamen University, School of Medicine, Xiamen University, Xiamen, China; ^2^Department of Hematology, Zhongshan Hospital of Xiamen University, School of Medicine, Xiamen University, Xiamen, China, China; ^3^Department of Epidemiology and Biostatistics, School of Public Health, Wuhan University, Wuhan, Hubei, China; ^4^Xiamen Cardiovascular Hospital of Xiamen University, School of Medicine, Xiamen University, Xiamen, China

**Keywords:** cardiovascular diseases, mortality, dietary risks, high body mass index, nursing care, China, Pakistan

## Abstract

**Background:**

Modifiable risk factors are major drivers of cardiovascular disease (CVD). We aimed to determine the epidemiological trend and age-period-cohort effects on CVD burden attributable to dietary risks and high body mass index (BMI) across China and Pakistan from 1990 to 2019.

**Methods:**

Data on the all-ages and age-specific CVD burden, age-standardized CVD mortality and disability-adjusted life years (DALYs) rates were obtained from the Global Burden of Disease Study 2019. Joinpoint regression analysis was conducted to find temporal trends and age-period-cohort (APC) modeling was used to estimate age, period, and cohort effects on CVD burden.

**Results:**

Between 1990 and 2019, the all-ages CVD burden attributable to dietary risks and high BMI increased by ~2-3-fold in China and by 3-5-fold in Pakistan. The diet-related CVD age-standardized mortality rate (ASMR) and age-standardized disability-adjusted life years (DALYs) rate significantly decreased in China but increased in Pakistan. Both countries showed a marked increasing trend of CVD ASMR and the age-standardized DALYs rate attributable to high BMI. Taiwan in China showed a remarkable reduction in CVD burden. However, in Pakistan, all regions observed a significantly increasing trend of CVD burden attributable to modifiable risk factors. A higher risk ratio of premature CVD mortality (<70 years) was observed among Chinese attributable to high BMI and among Pakistani attributable to dietary risks. In China, early birth cohorts showed a higher risk ratio and recent birth cohorts experienced a lower risk ratio of CVD burden compared with Pakistan.

**Conclusion:**

In conclusion, dietary risks and high BMI caused a huge CVD burden across China and Pakistan.

## Introduction

Cardiovascular disease (CVD) caused 18.6 million deaths worldwide and is considered the leading cause of premature mortality in 2019. Cardiovascular diseases (CVDs), particularly ischemic heart disease (IHD) and stroke accounted for 9.1 million and 6.5 million deaths in the year 2019, respectively (1). By 2030, CVD-related deaths would be more than 23 million across the globe (2). World Health Organization (WHO) estimated that more than three-quarters of CVDs deaths occurred in low- and middle-income countries (LMICs) which is considered a growing epidemic problem (3). Among LMICs, China and Pakistan are home to 1.45 billion and 0.23 billion people and ranked first and fifth with the largest population in 2022 in the world, respectively (4). Both China and Pakistan experiencing an increasing trend of CVD burden (5). The number of CVD deaths increased in China (2.42 million to 4.58 million) and Pakistan (0.17 million to 0.34 million) from 1990 to 2019 (1).

The huge increasing CVD burden in China and Pakistan could be attributed to several modifiable risk factors (1). The United Nations set a target to reduce premature CVD mortality by 25% at the end of 2025 attributable to behavioral and biological risk factors (6). Behavioral and metabolic risk factors such as dietary risks and high body mass index (BMI) are major drivers of CVD. In the GBD study 2019, dietary risks are either an over-consumed diet (sodium, trans-fatty acids, sugar-sweetened beverages, red meat, and processed meat) or an under-consumed diet (whole grains, legumes, vegetables, fruits, nuts and seeds, milk, fiber, calcium, omega-3 fatty acids from seafood, and polyunsaturated fatty acids) (1, 7). High BMI (≥25 kg/m^2^) is considered an epidemic worldwide. High BMI exacerbates CVD risk factors including blood pressure, lipids, blood sugars, and inflammation, and has a linear association with coronary heart diseases. Globally, dietary risks and high BMI caused 6.8 million and 3.2 million deaths and 153 million and 86 million disability-adjusted life years (DALYs) in 2019, respectively (1).

Several epidemiological studies reported a temporal trend of CVD attributable to smoking, low physical activity, air pollution, and dietary risks in the high socio-demographic index (SDI) and low SDI countries from 1990 to 2017 (8–10). However, no or limited studies (1, 5, 7, 11, 12) observed the recent times trends and age-period-cohort effects on CVD burden attributable to the dietary risk and high BMI at the regional and country level across China and Pakistan. In the age-period-cohort analysis, period and cohort effects would assist public health policymakers to determine the success of earlier health-policy interventions and identifying future targets (13). To provide advice and references for health policymakers, an accurate and comparable analysis of long-term CVD trends at the regional and country level is required. Therefore, we aimed to determine the epidemiological trend and age-period-cohort effect on CVD burden attributable to dietary risks and high BMI across China and Pakistan from 1990 to 2019.

## Materials and methods

### Data source

In this study, the data were extracted by sex (male, female, and both sex combined) from the global burden of diseases (GBD) free online database (GBD 2019)^1^ (14) (accessed on July 7, 2022) from 1990 to 2019. In addition, all-ages deaths, the age-standardized mortality rate (ASMR), and age-specific data (i.e., from 25–29 years to 85–89 years) on CVD mortality and disability-adjusted life years (DALYs) attributable to dietary risks and high BMI were extracted. GBD is an international cooperative project that estimates the disease burden at regional, national, and global levels. GDB estimates the burden of disease indices including, prevalence, incidence, mortality rate, years of life lost (YLL), years lived with disability (YLD), and DALYs for several diseases and injuries. Moreover, the GBD data are provided by different organizations like World Bank Open Data, WHO, and Global Health Observatory for different political and social research. The GBD data is managed by the Institute for Health Metrics and Evaluation (IHME), University of Washington. Therefore, a waiver of informed consent was reviewed and approved by the University of Washington Institutional Review Board (15, 16).

### Variables understudy

In the present study, the considered modifiable risk factors were dietary risks and high BMI (≥25 kg/m^2^). The dietary risk factor was a composite of an over-consumed diet (sodium, trans-fatty acids, sugar-sweetened beverages, red meat, and processed meat) and an under-consumed diet (whole grains, legumes, vegetables, fruits, nuts and seeds, milk, fiber, calcium, omega-3 fatty acids from seafood, and polyunsaturated fatty acids) (1). The outcome variables were ASMR, all-ages death numbers, and DALYs of CVD, ischemic heart disease (IHD), and ischemic stroke (IS) for China and Pakistan at the regional and country level from 1990 to 2019. Regions in Pakistan were Islamabad, Punjab, Sindh, Khyber Pakhtunkhwa (KPK), Balochistan, Azad Jammu & Kashmir (AJ& K), Gilgit-Baltistan (GB), and Taiwan in China. DALYs are defined as the sum of years lived with disability (YLDs) and years of life lost (YLLs) (16).

### Statistical analysis

#### Joinpoint regression for trend analysis (1990–2019)

To assess the temporal trends of CVD, IHD, and IS burden, we estimated the average annual percentage change (AAPC) for CVD, IHD, and IS mortality and DALYs with joinpoint regression analysis. AAPC represents the trend of CVD, IHD, and IS burden in the whole period from 1990 to 2019. Additionally, AAPC is a weighted average of the yearly percentage change determined by the joinpoint model, with weights corresponding to the duration of the annual percentage change (APC) interval. The APC shows the CVD, IHD, and IS burden trend in each segment determined by using joinpoint regression software. From 1990 to 2019, we produced AAPCs and their 95% confidence intervals (CIs) for each trend segment identified by the model. Furthermore, we estimated AAPCs of CVD, IHD, and IS deaths for both sexes combined, males, and females. AAPC is considered significant when it is different from 0 at the alpha of 0.05. This analysis was conducted using the joinpoint regression program version 4.9.1.0 (April 2022) from the Surveillance Research Program of the U.S. National Cancer Institute (NCI).

#### Age-period-cohort analysis

The aim of the age-period-cohort (APC) analysis is to estimate the effects of age, period, and cohort on CVD burden attributable to modifiable risk factors. The age effect represents the association of CVD burden with different age groups. Period effect represents influencing factors, such as a series of historical events and environmental factors, and it reflects variation in the CVD burden over time that influences all age groups simultaneously. The cohort effect shows variations of CVD burden across birth cohorts born in the same year and changes in different lifestyles (17). The common problem associated with the APC analysis is collinearity (i.e., birth cohort = period - age). The APC model is affected by the linearity between two variables, so it is impossible to determine the three independent linear APC variables of age, period, and cohort. We used the APC model with the intrinsic estimator (IE), which is a new method to estimate the coefficients and solve the collinearity problem by generating a distinctive set of trend estimates independent of any arbitrary assignment of identifying limitations on age, period, or cohort coefficients that may not be verified in the data itself (18). Estimated coefficients for the age, period, and cohort effects were produced by the APC analysis using the IE method. The exponential value [exp(coef.) = ecoef.] was created from these coefficients, which denotes the risk ratio (RR) of a particular age, period, or birth cohort relative to the reference group.

In the APC model using the IE method, the age-specific CVD rates were appropriately categorized into 13 age groups (from 25–29 years to 85–89 years). It has 6 periods with 5-year intervals (from 1990–1994 to 2015–2019) and 18 birth cohorts (period-age) (from1905-1909 to 1990–1994). The general form of the APC model is written as Y = log (M) = μ + αage_1_+ βperiod_1_ + γcohort_1_ + ε; where, M is defined as the incidence rate in the age groups, α, β, and γ indicates the functions of age, period, and cohort effect, μ, and ε are the intercept item and the random error. The APC model was used to decompose the three trends and estimate efficient results (19). Moreover, the Akaike information criterion (AIC), and Bayesian information criterion (BIC) were used to estimate and analyze the degree of fitting of the model. The APC analysis was done using Stata 15.0 software (College Station, TX, USA).

## Results

### CVD burden attributable to dietary risks

**Figure 1:**
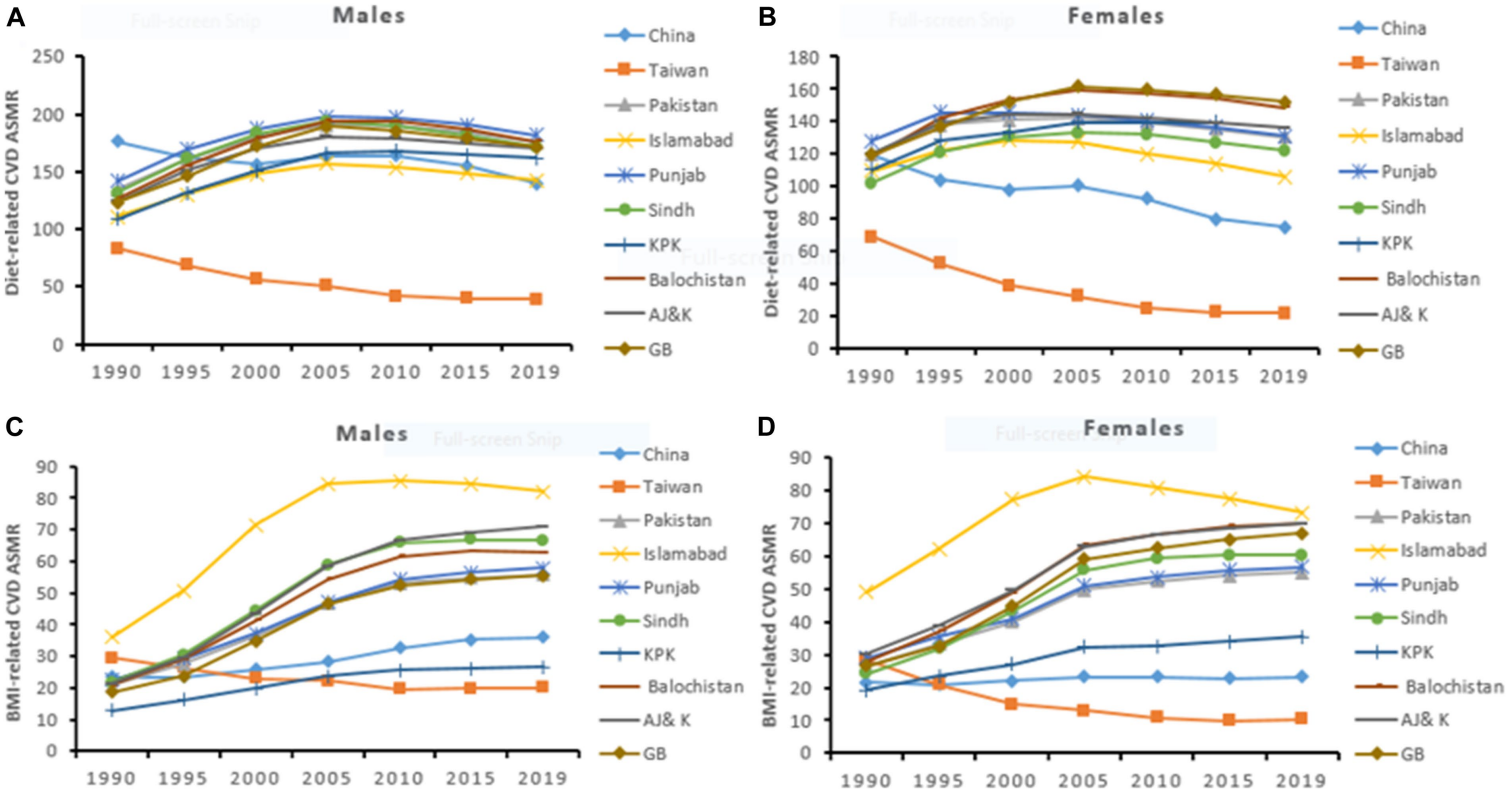
Temporal trend of CVD ASMR (per 100,000) attributable to dietary risks **(A,B)** and high BMI **(C,D)** in males and females across China and Pakistan.

For both sexes, the all-ages CVD deaths due to dietary risks significantly increased in China (0.9 million to 1.7 million) and in Pakistan (0.06 million to 0.15 million) by 89 and 150%, respectively during the study period. The CVD ASMR and the age-standardized DALYs rate in China significantly decreased by −1.2% (95%CI: −1.5, −0.9) and by −1.4% (95%CI: −1.7, −1.2) per year. However, in Pakistan, the ASMR of CVD and the age-standardized DALYs rate significantly increased by 0.7% (95%CI: 0.6, 0.7) and by 0.7% (95%CI: 0.6, 0.8) per year. At the regional level in China, Taiwan showed a remarkable reduction in CVD ASMR by −3.2% (95%CI: −3.5, −2.9) and age-standardized DALYs rate by −2.9% (95%CI, −3.1, −2.7). However, in Pakistan, all regions observed significantly increasing trends of CVD ASMR and age-standardized DALYs rate with the highest in KPK. Overall, male showed less improvement in CVD, IHD, and IS ASMR and age-standardized DALYs rate attributable to dietary risks than the female population across China and Pakistan (Tables 1–3; Supplementary Tables S1–S6; Figure 1; Supplementary Figure S1).

**Table 1:** The temporal trend in the burden of CVD mortality attributable to the dietary risk and high BMI for both sexes across China and Pakistan from 1990 to 2019.

Dietary risks	ASMR/100,000			Deaths, *n* × 10,000		
CVD	1990 (95%UI)	2019 (95%UI)	AAPC (95%CI)	1990 (95%UI)	2019 (95%UI)	AAPC (95%CI)
China	143 (185, 107)	101 (132, 74)	−1.2 (−1.5, −0.9)	99 (126, 74)	176 (230, 130)	2.0 (1.7, 2.3)
Taiwan	76 (102, 54)	29 (42, 19)	−3.2 (−3.5, −2.9)	0.9 (1.3, 0.7)	1.2 (1.7, 0.7)	0.6 (0.3, 0.9)
Pakistan	127 (159, 98)	153 (193, 121)	0.7 (0.6, 0.7)	6.7 (8.3, 5.2)	15 (19, 12)	2.9 (2.7, 3.1)
Islamabad	110 (143, 79)	125 (161, 92)	0.5 (0.4, 0.6)	0.02 (0.03, 0.01)	0.08 (0.1, 0.06)	4.7 (4.6, 4.8)
Punjab	135 (169, 104)	156 (201, 122)	0.5 (0.5, 0.6)	4.2 (5.3, 3.3)	8.9 (11.4, 6.9)	2.6 (2.4, 2.7)
Sindh	117 (152, 85)	147 (193, 110)	0.8 (0.7, 0.9)	1.2 (1.5, 0.8)	3.1 (4.1, 2.2)	3.4 (3.4, 3.5)
KPK	109 (151, 76)	150 (201, 106)	1.1 (1.0, 1.2)	0.8 (1.1, 0.5)	2.2 (3.1, 1.5)	3.5 (3.3, 3.6)
Balochistan	122 (165, 86)	162 (217, 119)	1.0 (0.9, 1.1)	0.2 (0.3, 0.1)	0.6 (0.8, 0.4)	3.1 (3.0, 3.2)
AJ&K	122 (163, 90)	152 (197, 111)	0.8 (0.7, 0.9)	0.2 (0.2, 0.1)	0.3 (0.4, 0.2)	2.6 (2.5, 2.7)
GB	121 (167, 85)	161 (220, 112)	1.0 (0.8, 1.2)	0.03 (0.04, 0.02)	0.1 (0.1, 0.07)	4.3 (4.2, 4.5)
High BMI						
China	22 (50, 5)	29 (51, 12)	0.9 (0.4, 1.4)	17 (38, 4)	54 (94, 24)	4.0 (3.6, 4.3)
Taiwan	29 (47, 12)	15 (24, 7)	−2.3 (−2.6, −2.0)	0.4 (0.7, 0.2)	0.5 (0.9, 0.2)	1.0 (0.7, 1.3)
Pakistan	23 (49, 6)	55 (87, 29)	3.1 (2.9, 3.2)	1.3 (2.7, 0.3)	6.3 (9.8, 3.4)	5.5 (5.4, 5.7)
Islamabad	41 (68, 20)	77 (110, 50)	2.2 (2.1, 2.4)	0.01 (0.01, 0.004)	0.06 (0.09, 0.04)	6.6 (6.4, 6.8)
Punjab	25 (52, 6)	57 (89, 30)	2.9 (2.7, 3.1)	0.8 (1.7, 0.2)	3.6 (5.7, 1.9)	5.2 (5.0, 5.4)
Sindh	22 (48, 6)	63 (99, 36)	3.7 (3.5, 3.8)	0.2 (0.5, 0.1)	1.5 (2.3, 0.8)	6.5 (6.4, 6.6)
KPK	15 (37, 3)	30 (63, 9)	2.4 (2.2, 2.5)	0.1 (0.3, 0.1)	0.5 (1.1, 0.1)	4.8 (4.6, 5.0)
Balochistan	23 (51, 6)	65 (106, 34)	3.6 (3.4, 3.7)	0.1 (0.1, 0.01)	0.3 (0.5, 0.1)	5.9 (5.7, 6.0)
AJ& K	25 (51, 7)	70 (107, 40)	3.6 (3.5, 3.8)	0.03 (0.06, 0.01)	0.1 (0.2, 0.09)	5.7 (5.6, 5.8)
GB	22 (51, 5)	61 (101, 29)	3.5 (3.4, 3.6)	0.01 (0.01, 0.001)	0.05 (0.08, 0.02)	6.9 (6.8, 7.1)

KPK, Khyber Pakhtunkhwa; AJ&K, Azad Jammu & Kashmir; GB, Gilgit Baltistan; CVD, cardiovascular disease; ASMR, age-standardized mortality rate; BMI, body mass index; AAPC, average annual percent change.

**Table 2:** The temporal trend in the burden of CVD DALYs attributable to the dietary risk and high BMI for both sexes across China and Pakistan from 1990 to 2019.

Dietary risks	Age-standardized DALYs/100,000			DALYs, *n* × 10,000		
CVD	1990 (95%UI)	2019 (95%UI)	AAPC (95%CI)	1990 (95%UI)	2019 (95%UI)	AAPC (95%CI)
China	3,061 (3,839, 2,345)	2,011 (2,572, 1,505)	−1.4 (−1.7, −1.2)	2,604 (3,242, 2008)	3,904 (4,970, 2,918)	1.5 (1.2, 1.7)
Taiwan	1,556 (2045, 1,133)	660 (917, 456)	−2.9 (−3.1, −2.7)	24 (31, 17)	25 (35, 17)	0.1 (−0.1, 0.3)
Pakistan	2,950 (3,625, 2,321)	3,602 (4,516, 2,839)	0.7 (0.6, 0.8)	177 (216, 140)	450 (564, 354)	3.3 (3.2, 3.4)
Islamabad	2,387 (3,126, 1,680)	2,650 (3,541, 1888)	0.4 (0.3, 0.5)	0.6 (0.8, 0.4)	2.5 (3.5, 1.6)	5.0 (4.8, 5.1)
Punjab	3,154 (3,918, 2,471)	3,691 (4,708, 2,868)	0.6 (0.5, 0.6)	109 (135, 86)	258 (331, 200)	3.0 (2.9, 3.1)
Sindh	2,700 (3,493, 1977)	3,425 (4,515, 2,547)	0.9 (0.8, 1.0)	31 (41, 23)	91 (122, 67)	3.8 (3.7, 3.9)
KPK	2,551 (3,514, 1810)	3,525 (4,751, 2,514)	1.1 (1.0, 1.2)	22 (30, 15)	64 (87, 45)	3.7 (3.5, 3.9)
Balochistan	2,863 (3,926, 1965)	3,880 (5,208, 2,796)	1.1 (1.0, 1.2)	7 (10, 5)	20 (27, 14)	3.5 (3.4, 3.6)
AJ&K	2,798 (3,764, 2082)	3,422 (4,519, 2,500)	0.7 (0.6, 0.8)	3.9 (5.2, 2.9)	8.9 (11, 6.4)	2.9 (2.8, 3.0)
GB	2,877 (4,004, 2003)	3,881 (5,319, 2,687)	1.1 (0.9, 1.3)	1.0 (1.4, 0.6)	3.5 (4.9, 2.4)	4.5 (4.3, 4.7)
High BMI						
China	576 (1,251, 141)	747 (1,253, 343)	0.9 (0.5, 1.3)	532 (1,129, 132)	1,500 (2,494, 694)	3.6 (3.3, 4.0)
Taiwan	744 (1,188, 342)	450 (699, 247)	−1.7 (−2.0, −1.5)	12 (19, 5)	16 (25, 9)	0.9 (0.7, 1.2)
Pakistan	642 (1,325, 176)	1,539 (2,384, 842)	3.1 (3.0, 3.3)	40 (83, 11)	206 (317, 113)	5.8 (5.6, 6.0)
Islamabad	1,093 (1,804, 540)	1,955 (2,787, 1,269)	2.1 (1.9, 2.2)	0.3 (0.5, 0.1)	2.1 (3.1, 1.2)	6.8 (6.5, 7.1)
Punjab	698 (1,436, 190)	1,601 (2,467, 862)	3.0 (2.8, 3.1)	25 (51, 6)	119 (184, 65)	5.6 (5.4, 5.7)
Sindh	617 (1,276, 171)	1,740 (2,683, 998)	3.7 (3.5, 3.9)	7.7 (16, 2.1)	50 (77, 28)	6.7 (6.6, 6.9)
KPK	440 (1,050, 86)	853 (1,717, 261)	2.3 (2.1, 2.5)	4.1 (9.7, 0.8)	16 (33, 5)	4.9 (4.8, 5.1)
Balochistan	658 (1,421, 175)	1,839 (2,988, 966)	3.7 (3.4, 3.9)	1.8 (4.1, 0.4)	10 (17, 5)	6.2 (6.1, 6.3)
AJ&K	688 (1,412, 203)	1,878 (2,845, 1,112)	3.6 (3.3, 3.9)	1.1 (2.1, 0.2)	5.3 (8.1, 3.1)	5.9 (5.8, 6.0)
GB	629 (1,417, 152)	1,721 (2,866, 842)	3.6 (3.5, 3.7)	0.2 (0.5, 0.1)	1.7 (2.8, 0.8)	7.1 (7.0, 7.2)

KPK, Khyber Pakhtunkhwa; AJ&K, Azad Jammu & Kashmir; GB, Gilgit Baltistan; CVD, cardiovascular disease; DALYs, disability-adjusted life years; BMI, body mass index; AAPC, average annual percent change.

**Table 3:** The temporal trend in the burden of CVD AMSR and DALYs attributable to the dietary risk and high BMI in males and females across China and Pakistan from 1990 to 2019.

CVD ASMR	Male (AAPC) (95%CI)		Female (AAPC) (95%CI)	
	Dietary risks	High BMI	Dietary risks	High BMI
China	−0.8 (−1.1, −0.5)	1.5 (1.3, 1.7)	−1.7 (−1.9, −1.4)	0.2 (−0.1, 0.6)
Taiwan	−2.6 (−2.9, −2.2)	−1.3 (−1.7, −1.0)	−3.9 (−4.2, −3.6)	−3.4 (−3.7, −3.2)
Pakistan	1.0 (0.9, 1.1)	3.5 (3.4, 3.7)	0.3 (0.3, 0.4)	2.6 (2.4, 2.7)
Islamabad	0.9 (0.8, 1.0)	3.0 (2.7, 3.2)	−0.1 (−0.2, −0.1)	1.4 (1.3, 1.6)
Punjab	0.9 (0.8, 1.0)	3.4 (3.3, 3.5)	0.1 (0.1, 0.2)	2.4 (2.3, 2.5)
Sindh	1.0 (0.9, 1.1)	4.0 (3.8, 4.2)	0.6 (0.6, 0.7)	3.2 (3.1, 3.3)
KPK	1.4 (1.3, 1.5)	2.6 (2.4, 2.7)	0.8 (0.7, 0.9)	2.1 (1.9, 2.3)
Balochistan	1.2 (1.1, 1.3)	4.0 (3.7, 4.3)	0.8 (0.7, 0.9)	3.3 (3.1, 3.4)
AJ&K	1.1 (1.0, 1.2)	4.2 (4.0, 4.4)	0.4 (0.4, 0.5)	3.0 (2.8, 3.1)
GB	1.2 (1.0, 1.4)	3.9 (3.8, 4.0)	0.9 (0.7, 1.1)	3.3 (3.1, 3.4)
CVD DALYs				
China	−1.0 (−1.3, −0.7)	1.5 (1.2, 1.9)	−2.1 (−2.3, −1.8)	0.1 (−0.2, 0.2)
Taiwan	−2.3 (−2.6, −2.0)	−0.9 (−1.4, −0.5)	−3.7 (−3.9, −3.5)	−2.8 (−3.0, −2.6)
Pakistan	1.1 (1.0, 1.2)	3.6 (3.4, 3.8)	0.3 (0.2, 0.3)	2.6 (2.4, 2.7)
Islamabad	0.8 (0.7, 1.0)	2.8 (2.4, 3.1)	−0.3 (−0.4, −0.1)	1.2 (1.0, 1.4)
Punjab	1.0 (0.9, 1.1)	3.4 (3.3, 3.6)	0.1 (−0.1, 0.1)	2.5 (2.2, 2.7)
Sindh	1.1 (1.0, 1.2)	4.0 (3.8, 4.3)	0.6 (0.5, 0.6)	3.3 (3.1, 3.4)
KPK	1.5 (1.4, 1.7)	2.6 (2.5, 2.8)	0.7 (0.6, 0.8)	2.1 (1.9, 2.3)
Balochistan	1.4 (1.2, 1.5)	4.1 (3.8, 4.4)	0.8 (0.7, 0.9)	3.3 (3.1, 3.4)
AJ& K	1.2 (1.0, 1.3)	4.2 (4.0, 4.4)	0.3 (0.2, 0.4)	2.9 (2.7, 3.1)
GB	1.3 (1.2, 1.5)	4.0 (3.9, 4.1)	0.8 (0.6, 1.0)	3.2 (3.1, 3.4)

KPK, Khyber Pakhtunkhwa; AJ&K, Azad Jammu & Kashmir; GB, Gilgit Baltistan; CVD, cardiovascular disease; ASMR, age-standardized mortality rate; DALYs, disability-adjusted life years; BMI, body mass index; AAPC, average annual percent change.

### CVD burden attributable to high BMI

For both sexes, the all ages-CVD deaths attributable to high BMI remarkably increased in China by 4.0% (95%CI: 3.6, 4.3) and in Pakistan by 5.5% (95%CI: 5.4, 5.7) per year. Similarly, the ASMR of CVD and the age-standardized DALYs rate significantly increased in both China and Pakistan. At the regional level in China, Taiwan experienced a pronounced reduction in CVD ASMR by −2.3% (95%CI: −2.6, −2.0) and age-standardized DALYs rate by −1.7 (95%CI: −2.0, −1.5) per year. On the other hand, in Pakistan, all regions showed a significantly increasing trend of CVD ASMR and age-standardized DALYs rates notably in Sindh, Balochistan, and AJ&K. Moreover, Islamabad experienced higher CVD ASMR and DALYs rates during the study period. The male population showed the fastest increasing trend of CVD, IHD, and IS ASMR and age-standardized DALYs rate than the female population in both countries (Tables 1–3; Supplementary Tables S1–S6; Figure 1; Supplementary Figure S1).

### Age-period-cohort effect on CVD burden attributable to dietary risks and high BMI

**Figure 2:**
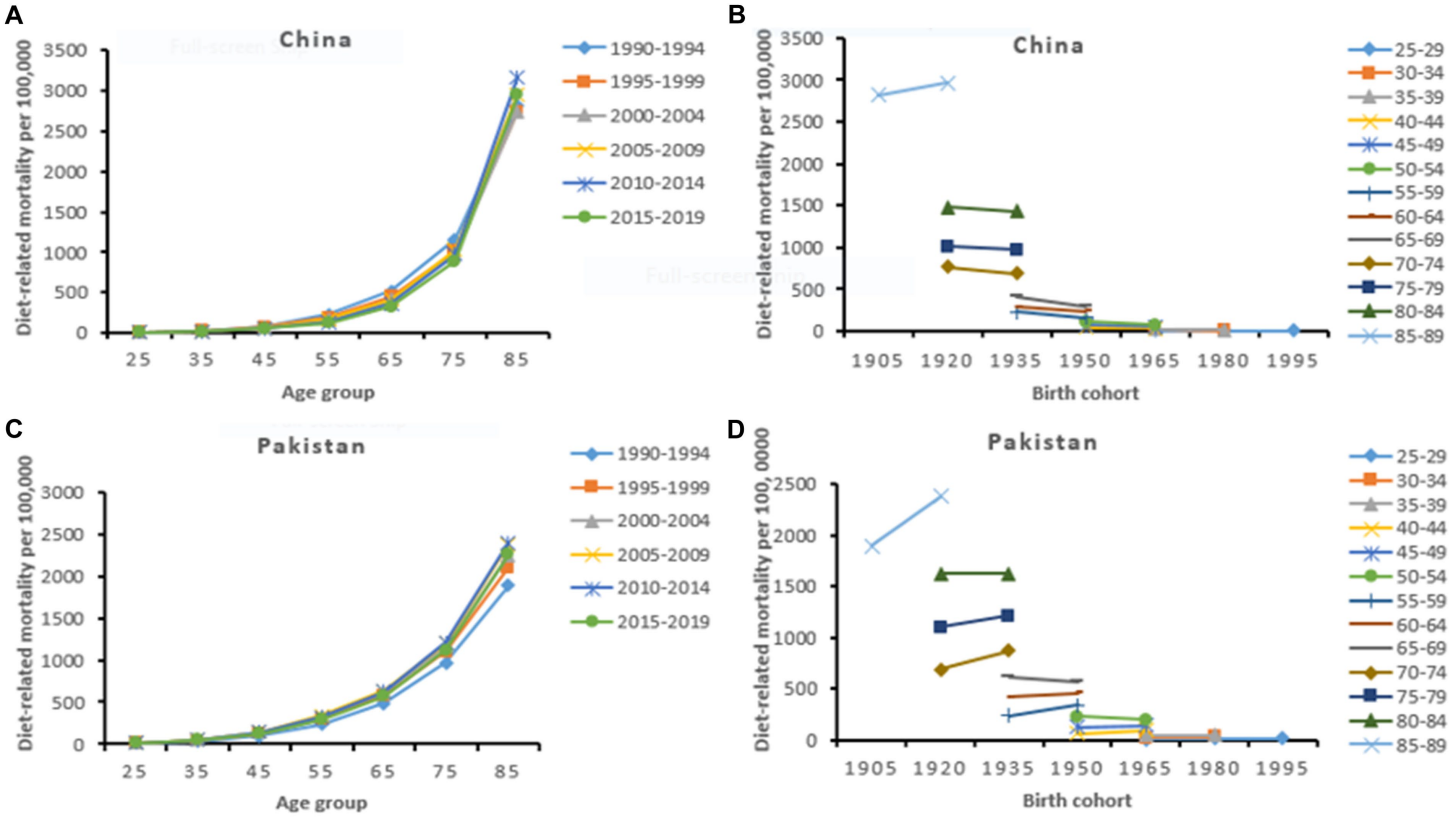
Diet-related age-specific CVD mortality rate by period **(A,C)** and cohort-specific CVD mortality rate by age group **(B,D)** across China and Pakistan from 1990 to 2019.

**Figure 3:**
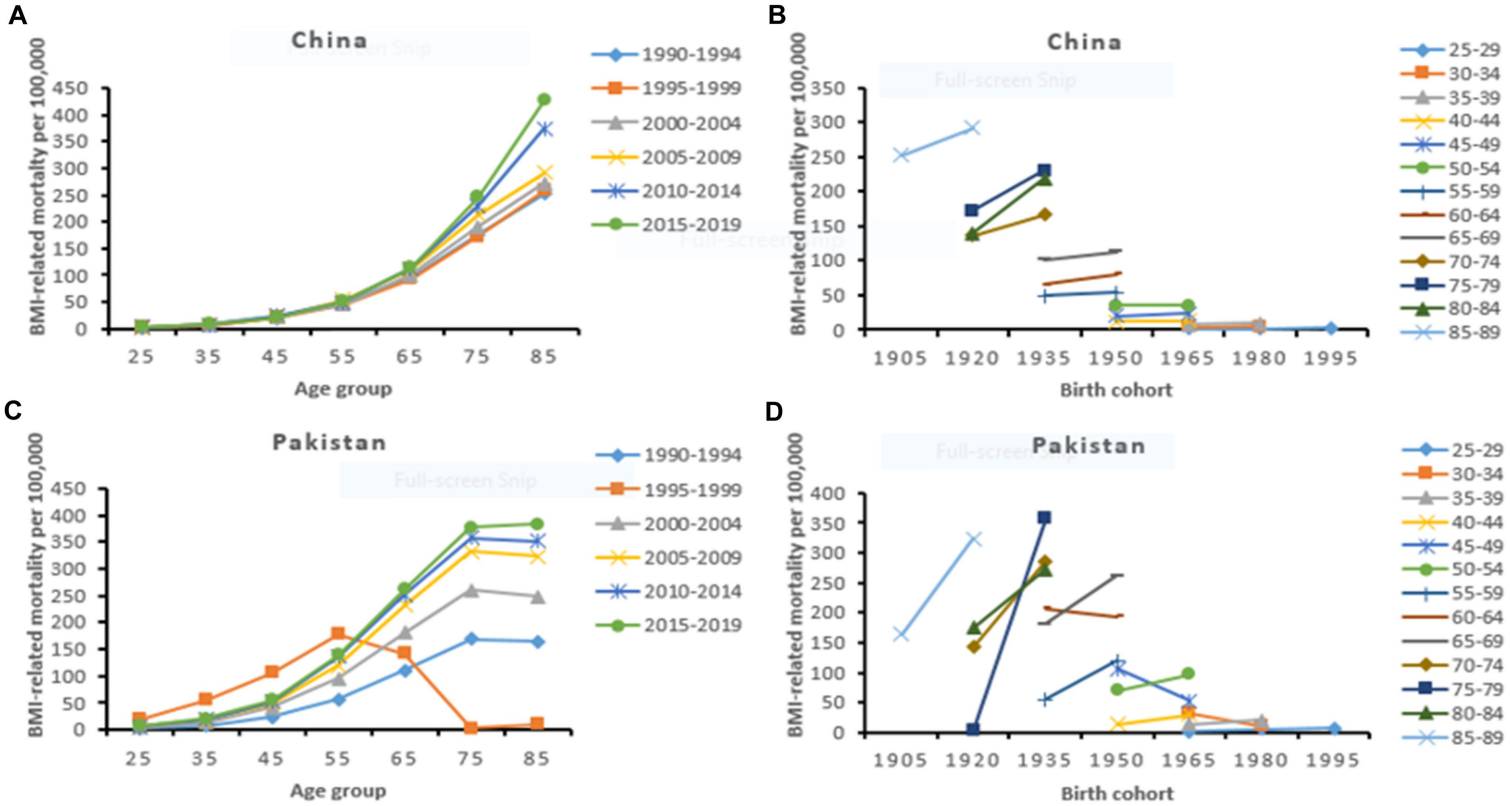
High BMI-related age-specific CVD mortality rate by period **(A,C)** and cohort-specific CVD mortality rate by age group **(B,D)** across China and Pakistan from 1990 to 2019.

The risk ratio of CVD mortality and DALYs due to dietary risks and high BMI markedly increased with age. China had the highest risk of CVD mortality across all age groups, particularly at older ages than Pakistan. The risk ratio of premature CVD mortality (<70 years) attributable to dietary risks was higher among Pakistanis. However, the Chinese population showed a higher risk ratio of premature CVD mortality attributable to high BMI. Period effects were generally higher for CVD mortality and DALYs attributable to high BMI with the most remarkable in Pakistan. Compared to the reference cohorts, the risk ratio of cohort effect on CVD burden attributable to dietary risks and high BMI showed a similar downward trend among the Chinese and Pakistani populations. Early birth cohorts in China had a higher risk ratio of CVD mortality and DALYs than in Pakistan. However, recent birth cohorts in China showed relatively a lower risk ratio of CVD burden attributable to modifiable risk factors (Tables 4, 5; Figures 2, 3; Supplementary Figures S2, S3).

**Table 4:** Age-Period-Cohort effects on CVD mortality attributable to dietary risks and high BMI across China and Pakistan.

Variables	China CVD mortality (RR 95% CI)		Pakistan CVD mortality (RR 95% CI)	
	Dietary risks	High BMI	Dietary risks	High BMI
Age				
25–29	1.00	1.00	1.00	1.00
30–34	1.65 (1.65, 149)	1.74 (2.13, 1.49)	1.93 (2.07, 1.79)	1.46 (1.58, 1.35)
35–39	2.72 (3.01, 2.23)	2.73 (3.56, 2.16)	3.12 (3.46, 2.81)	1.97 (2.20, 1.75)
40–44	4.06 (4.97, 3.32)	4.60 (6.38, 3.46)	5.05 (5.75, 4.45)	2.56 (2.94, 2.22)
45–49	5.48 (6.71, 4.96)	6.47 (9.30, 4.65)	7.21 (8.32, 6.27)	3.27 (3.84, 2.78)
50–54	8.18 (10.01, 6.70)	9.20 (13.79, 6.44)	10.39 (12.16, 8.91)	4.56 (5.48, 3.80)
55–59	10.40 (13.11, 8.18)	11.21 (17.35, 7.72)	13.42 (15.87, 11.39)	5.49 (6.68, 4.52)
60–64	13.49 (16.50, 11.05)	14.60 (23.00, 9.72)	16.93 (20.20, 14.22)	6.95 (8.56, 5.65)
65–69	18.22 (24.62, 14.91)	18.77 (30.19, 12.25)	20.78 (25.01, 17.32)	7.79 (9.68, 6.29)
70–74	30.04 (33.23, 22.25)	25.72 (42.29, 16.47)	25.84 (31.28, 21.43)	9.76 (12.19, 7.82)
75–79	36.69 (44.86, 27.17)	30.79 (50.43, 19.72)	32.17 (39.02, 26.60)	10.02 (12.48, 8.04)
80–84	54.73 (66.93, 40.54)	22.88 (37.07, 14.76)	40.85 (49.45, 33.88)	7.59 (9.36, 6.17)
85–89	90.25 (121.95, 73.88)	40.74 (65.40, 26.62)	50.65 (60.83, 42.26)	11.41 (14.01, 9.30)
Period				
1990–1994	1.00	1.00	1.00	1.00
1995–1999	0.95 (0.95, 0.92)	1.04 (1.06, 1.02)	1.29 (1.30, 1.28)	1.30 (1.32, 1.29)
2000–2004	0.97 (0.99, 0.94)	1.21 (1.25, 1.17)	1.54 (1.56, 1.51)	1.52 (1.55, 1.49)
2005–2009	1.10 (1.12, 1.07)	1.43 (1.47, 1.38)	1.79 (1.81, 1.76)	1.95 (2.01, 1.91)
2010–2014	1.19 (1.22, 1.18)	1.74 (1.78, 1.70)	1.98 (2.01, 1.96)	2.13 (2.18, 2.09)
2015–2019	1.12 (1.14, 1.13)	2.08 (2.10, 2.06)	2.06 (2.07, 2.05)	2.42 (2.46, 2.38)
Cohort				
1905–1909	1.00	1.00	1.00	1.00
1910–1914	1.05 (1.06, 1.03)	0.99 (1.03, 0.96)	0.87 (0.88, 0.85)	0.39 (0.39, 0.39)
1915–1919	1.02 (1.04, 0.98)	0.90 (0.96, 0.85)	0.77 (0.79, 0.75)	0.78 (0.83, 0.74)
1920–1924	0.95 (0.98, 0.91)	0.83 (0.89, 0.77)	0.70 (0.72, 0.67)	0.78 (0.84, 0.73)
1925–1929	0.92 (0.95, 0.87)	0.82 (0.90, 0.75)	0.63 (0.65, 0.60)	1.05 (1.15, 0.96)
1930–1934	0.84 (0.86, 0.80)	0.79 (0.86, 0.72)	0.56 (0.58, 0.54)	1.02 (1.12, 0.93)
1935–1939	0.75 (0.77, 0.72)	0.71 (0.77, 0.66)	0.50 (0.52, 0.49)	1.10 (1.20, 1.01)
1940–1944	0.64 (0.65, 0.63)	0.62 (0.65, 0.58)	0.45 (0.46, 0.44)	1.06 (1.15, 0.98)
1945–1949	0.54 (0.54, 0.54)	0.54 (0.56, 0.52)	0.40 (0.41, 0.40)	0.97 (1.04, 0.91)
1950–1954	0.47 (0.48, 0.46)	0.49 (0.49, 0.49)	0.36 (0.36, 0.36)	0.91 (0.96, 0.86)
1955–1959	0.39 (0.40, 0.37)	0.42 (0.42, 0.41)	0.32 (0.32, 0.31)	0.79 (0.82, 0.76)
1960–1964	0.32 (0.33, 0.30)	0.35 (0.37, 0.33)	0.28 (0.29, 0.27)	0.68 (0.70, 0.66)
1965–1969	0.28 (0.30, 0.26)	0.32 (0.34, 0.29)	0.25 (0.26, 0.24)	0.54 (0.55, 0.54)
1970–1974	0.24 (0.27, 0.22)	0.28 (0.32, 0.24)	0.23 (0.24, 0.21)	0.44 (0.45, 0.43)
1975–1979	0.21 (0.45, 0.24)	0.25 (0.31, 0.21)	0.21 (0.22, 0.19)	0.32 (0.35, 0.30)
1980–1984	0.19 (0.24, 0.15)	0.25 (0.33, 0.21)	0.19 (0.22, 0.17)	0.26 (0.30, 0.23)
1985–1989	0.17 (0.24, 0.11)	0.23 (0.37, 0.14)	0.18 (0.22, 0.15)	0.22 (0.29, 0.18)
1990–1994	0.14 (0.33, 0.06)	0.20 (0.59, 0.06)	0.18 (0.29, 0.11)	0.19 (0.34, 0.10)
AIC	7.90	6.45	8.36	20.63
BIC	−175.66	−184.16	−176.93	880.92

CVD, cardiovascular disease; BMI, body mass index; RR, risk ratio; AIC, Akaike information criterion; BIC, Bayesian information criterion.

**Table 5:** Age-Period-Cohort effects on CVD DALYs attributable to dietary risks and high BMI across China and Pakistan.

Variables	China CVD DALYs (RR 95% CI)		Pakistan CVD DALYs (RR 95% CI)	
	Dietary risks	High BMI	Dietary risks	High BMI
Age				
25–29	1.00	1.00	1.00	1.00
30–34	1.55 (1.56, 1.53)	1.66 (1.70, 1.63)	1.81 (1.83, 1.80)	1.68 (1.70, 1.65)
35–39	2.35 (2.38, 2.32)	2.43 (2.49, 2.37)	2.78 (2.81, 2.74)	2.40 (2.44, 2.35)
40–44	3.44 (3.50, 3.38)	3.81 (3.94, 3.69)	4.22 (4.28, 4.16)	3.65 (3.74, 3.57)
45–49	4.52 (4.62, 4.43)	5.01 (5.21, 4.83)	5.61 (5.70, 5.51)	4.99 (5.12, 4.86)
50–54	6.11 (6.26, 5.97)	6.66 (6.94, 6.39)	7.45 (7.59, 7.32)	7.26 (7.47, 7.05)
55–59	7.37 (7.56, 7.19)	7.53 (7.86, 7.20)	8.76 (8.93, 8.60)	8.31 (8.57, 8.07)
60–64	8.93 (9.18, 8.70)	8.74 (9.16, 8.35)	9.89 (10.08, 9.69)	9.75 (10.07, 9.44)
65–69	10.70 (11.01, 10.40)	9.85 (10.35, 9.39)	10.62 (10.85, 10.40)	10.36 (10.70, 10.01)
70–74	12.96 (13.37, 12.60)	11.44 (12.04, 10.89)	11.24 (11.49, 11.00)	10.07 (10.41, 9.74)
75–79	14.49 (14.93, 14.07)	11.18 (11.76, 10.63)	11.50 (11.76, 11.25)	8.81 (9.11, 8.52)
80–84	16.68 (17.19, 16.21)	6.58 (6.90, 6.28)	11.64 (11.89, 11.39)	4.52 (4.66, 4.38)
85–89	22.40 (23.06, 21.81)	9.21 (9.66, 8.79)	11.38 (11.61, 11.13)	4.60 (4.74, 4.47)
Period				
1990–1994	1.00	1.00	1.00	1.00
1995–1999	0.92 (0.92, 0.92)	1.03 (1.03, 1.03)	1.26 (1.27, 1.26)	1.34 (1.35, 1.34)
2000–2004	0.92 (0.92, 0.92)	1.18 (1.18, 1.17)	1.46 (1.46, 1.46)	1.79 (1.79, 1.78)
2005–2009	1.01 (1.01, 1.01)	1.35 (1.35, 1.34)	1.63 (1.63, 1.63)	2.39 (2.39, 2.38)
2010–2014	1.05 (1.05, 1.05)	1.56 (157, 1.56)	1.73 (1.73, 1.73)	2.75 (2.76, 2.74)
2015–2019	0.98 (0.98, 0.98)	1.81 (1.82, 1.81)	1.72 (1.72, 1.72)	3.05 (3.06, 3.04)
Cohort				
1905–1909	1.00	1.00	1.00	1.00
1910–1914	1.06 (1.07, 1.06)	1.01 (1.02, 1.01)	0.89 (0.89, 0.89)	0.90 (0.91, 0.89)
1915–1919	1.05 (1.06, 1.05)	0.94 (0.96, 0.93)	0.82 (0.83, 0.81)	0.85 (0.87, 0.83)
1920–1924	1.02 (1.03, 1.01)	0.89 (0.91, 0.87)	0.76 (0.77, 0.76)	0.80 (0.83, 0.78)
1925–1929	1.02 (1.03, 1.01)	0.91 (0.93,0.89)	0.71 (0.72, 0.70)	0.76 (0.78, 0.74)
1930–1934	0.97 (0.97, 0.96)	0.89 (0.91, 0.87)	0.66 (0.67, 0.66)	0.72 (0.75, 0.70)
1935–1939	0.90 (0.90, 0.89)	0.84 (0.86, 0.82)	0.61 (0.62, 0.61)	0.69 (0.71, 0.67)
1940–1944	0.80 (0.80, 0.79)	0.77 (0.78, 0.75)	0.57 (0.57, 0.57)	0.65 (0.67, 0.63)
1945–1949	0.70 (0.71, 0.70)	0.70 (0.71, 0.69)	0.53 (0.53, 0.53)	0.62 (0.64, 0.60)
1950–1954	0.64 (0.64, 0.64)	0.66 (0.67, 0.65)	0.49 (0.50, 0.49)	0.60 (0.61, 0.58)
1955–1959	0.55 (0.55, 0.55)	0.59 (0.59, 0.58)	0.45 (0.46, 0.45)	0.57 (0.58, 0.55)
1960–1964	0.47 (0.47, 0.47)	0.52 (0.52, 0.51)	0.42 (0.42, 0.42)	0.53 (0.54, 0.52)
1965–1969	0.44 (0.44, 0.43)	0.49 (0.49, 0.49)	0.39 (0.39, 0.39)	0.49 (0.50, 0.48)
1970–1974	0.39 (0.40, 0.39)	0.44 (0.44, 0.44)	0.37 (0.37, 0.36)	0.46 (0.47, 0.46)
1975–1979	0.35 (0.36, 0.35)	0.42 (0.43, 0.42)	0.35 (0.35, 0.35)	0.45 (0.46, 0.45)
1980–1984	0.33 (0.34, 0.32)	0.42 (0.43, 0.42)	0.34 (0.34, 0.33)	0.44 (0.44, 0.44)
1985–1989	0.30 (0.32, 0.29)	0.41 (0.43, 0.39)	0.33 (0.34, 0.33)	0.42 (0.43, 0.42)
1990–1994	0.27 (0.29, 0.24)	0.38 (0.42, 0.34)	0.34 (0.36, 0.32)	0.42 (0.44, 0.40)
AIC	16.74	11.99	16.25	11.08
BIC	246.48	−19.42	174.96	−128.28

CVD, cardiovascular disease; BMI, body mass index; DALYs, disability-adjusted life years; RR, risk ratio; AIC, Akaike information criterion; BIC, Bayesian information criterion.

## Discussion

The present GBD study 2019 provides the epidemiological trend (1990–2019) and age-period-cohort effect on CVD mortality and DALYs attributable to dietary risks and high BMI at the regional and country level across China and Pakistan. We observed that the all-ages CVD deaths and DALYs due to dietary risks and high BMI significantly increased in China and Pakistan during the study period. The diet-related CVD ASMR and the age-standardized DALYs rate significantly decreased in China. However, the ASMR of CVD and the age-standardized DALYs rate attributable to dietary risks significantly increased in Pakistan. Both countries showed a marked increasing trend of CVD ASMR and the age-standardized DALYs rate attributable to high BMI. At the regional level in China, Taiwan showed a remarkable reduction in CVD ASMR and age-standardized DALYs rate attributable to dietary risks and high BMI. However, in Pakistan, all regions observed significantly increasing trends of CVD ASMR and age-standardized DALYs rate attributable to aforementioned modifiable risk factors.

Age-period-cohort analysis revealed that the risk ratio of premature CVD mortality (<70 years) attributable to dietary risks was higher among Pakistanis. On the other hand, the Chinese population showed a higher risk ratio of premature CVD mortality attributable to high BMI. The risk ratio of period effect on CVD burden attributable to modifiable risk factors significantly increased across both countries. In China, early birth cohorts showed a higher risk and recent birth cohorts experienced a lower risk of CVD burden compared with Pakistan.

### Temporal trend in CVD burden attributable to dietary risks

CVDs are the primary consequences of dietary risks and one-third of global CVD deaths are attributed to dietary risks (1). Our study found that the all-ages CVD deaths and DALYs due to dietary risks nearly doubled in China and tripled in Pakistan. The upward trend in all-ages CVD burden could be explained by population growth and aging across China and Pakistan (1). Aging has a profound impact on heart health and the arterial system and has been linked with a higher risk of CVD (20). Globally, adults aged ≥65 years increased by 3-fold over the last four decades. The older population in China and Pakistan is increasing and is projected to double by 2050 (4). It suggests that both countries are needed to significantly invest in the primary and secondary prevention of CVD among the older population.

Our findings show that ASMR of CVD and the age-standardized DALYs rate attributable to dietary risks significantly decreased in China as well as at the regional level in Taiwan. The reduction in CVD burden attributable to dietary risks could be explained by improvements in diet quality and changes in diet patterns among the Chinese population. The diet quality as measured by China Dietary Guidelines Index 2018 (CDGI-2018) improved from 41.7 to 52.4 during 1991–2015 among Chinese adults aged 18 years or older. Energy intake from high-quality protein and high-quality fats significantly increased whereas consumption of low-quality carbohydrates significantly decreased (21). A lower risk of CVD mortality was observed among Chinese adults having higher diet index scores and optimal consumption of vegetables, fruits, and lower consumption of red meat (22).

Moreover, the increasing trend in urbanization, higher education level, and socioeconomic status have improved diet quality among the Chinese population and resulted in lower CVD deaths (23, 24). The most recently launched public health and nutrition-related policy “Healthy China Action Plan (2019–2030)” including the popularization of health knowledge and promotion of a balanced diet could further improve diet quality and reduce diet-related CVD mortality and morbidity (25). The diet quality and dietary habits in Taiwan significantly improved such as higher consumption of vegetables, fruits, whole grains, fish, soy products, nuts, and seeds (26, 27). These positive changes in dietary habits could explain the remarkable reduction in the CVD burden at the regional level in Taiwan.

We observed significantly increasing trends of CVD ASMR and age-standardized DALYs rate attributable to dietary risks in Pakistan and across all regions with the highest in KPK. Pakistan is a country with a low socioeconomic status (SES). Poverty and low SES affect diet quality and are associated with adverse health outcomes (28). Moreover, Pakistan is an agricultural country with a large population that lives in rural areas with a lower *per capita* income (31%) than in urban areas. One-fifth of the population is malnourished and one-third of the population has no access to adequate nutrition (28). All these interrelated factors could explain a marked increase in CVD burden attributable to dietary risks. A previous study also reported that the Pakistani traditional diet was associated with high cardiovascular risk factors (29). The majority of Pakistani people eat deep-frying foods and foods high in saturated fats which ultimately increased the risk of CVD (30).

Pakistan is also initiated public health and nutrition-related policy to promote health knowledge and awareness about healthy food choices as adopted by China (25). Over the last few years, several nutritional programs have been launched by the government and non-government organizations to raise nutritional awareness among the Pakistani population. The government of Pakistan has initiated health and nutrition programs at the school level to provide relevant nutritional information to students (31, 32). At the community level, Nutrition International (NI) works with the Pakistani government to combat micronutrient malnutrition such as deficiency of iron, zinc, folic acid, and vitamin A (32). In the coming years, these nutritional intervention and awareness programs will gradually decrease the CVD burden attributable to dietary risks among the Pakistani population.

### Temporal trend in CVD burden attributable to high BMI

Obesity or high BMI is a growing public health epidemic problem and its prevalence increased by 3-fold over the last four and half decades across the globe. High intake of energy-dense foods, lower energy expenditure, and low physical activity lead to obesity (1, 33). High BMI exacerbates cardiovascular risk factors and increased the risk of CVD including hypertension, heart failure, and coronary heart disease (34). We observed that the all-ages CVD deaths and DALYs attributable to high BMI increased by 3-fold in China and by 5-fold in Pakistan. Moreover, the all-ages IHD, IS deaths and DALYs increased by 5-fold in China and by ~6-fold across Pakistan. The ASMR and age-standardized DALYs rate across all CVD categories significantly increased in China and Pakistan. All regions in Pakistan observed significantly increasing trends of CVD ASMR and age-standardized DALYs rate attributable to high BMI notably in Sindh, Balochistan, and AJ& K. However, Taiwan in China showed a marked reduction in CVD ASMR and age-standardized DALYs rate due to high BMI.

High BMI or obesity is a serious public health problem in China. More than half of the adult population and one-fifth of children and adolescents are overweight or obese and the rates are continuously rising in China (35). The continuously increasing burden of obesity could be driven by rapid economic growth. It has reduced poverty and impacted dietary habits, moving away from a traditional and balanced diet and adhering to ultra-processed junk food product. Physical activity substantially decreased with an increasingly sedentary lifestyle in China (36, 37). Such changes in diet patterns and lifestyle and other risk factors including genetic susceptibility, and psychosocial factors are implicated in the increasing trend of obesity in China and associated with a higher risk of CVD (36, 37). In the general Chinese population, obesity was associated with higher odds of CVDs (38).

Over the last two decades, several prevention and interventional programs have been implemented in China, however, obesity and chronic diseases such as CVD have not been effectively prevented and reduced yet (39). Recently in 2020, the National Health Commission of the People’s Republic of China (China NHC) formulated a series of policies, guidelines, and recommendations, which aims to effectively control, prevent, and reduce obesity among Children and adolescents. Moreover, in 2021, the China NHC’s Bureau of Disease Control and Prevention released a set of guidelines for obesity prevention and control among Chinese adults (40). In 2022, “The Expert Consensus on Obesity Prevention and Treatment” was issued which consisted of prevention and control strategies, and future recommendations (39). It reflects that China has made a great effort for obesity prevention which will ultimately decrease obesity-related CVD burden in the recent future. However, if these obesity-related policies and interventions have not effectively implemented, it is projected that about 65.3% of adults and 31.8% of children and adolescents could become overweight and obese by 2030 in China (35, 40).

Our findings show that the CVD ASMR and age-standardized DALYs rate attributable to high BMI markedly increased in Pakistan and across all regions. Pakistan with 50% of obesity is the tenth most obese nation in the world due to intake of a high-fat diet and sedentary lifestyle (41, 42). Among the Pakistani population, diet mostly consisted of saturated fats and trans fatty acids. The trend of eating fast food, processed foods, and junk food increased among the young population. Moreover, rapid urbanization and modernization have shifted the intake of a traditional low-fat diet to a high-fat diet (43). According to a systematic review and meta-analysis, Pakistani adolescents had a low level of physical activity and less than one-fifth has achieved recommended levels (44). The higher consumption of high-fat and saturated-fat diets, and low physical activity could be the contributing factors to increasing obesity which resulted in high CVD mortality and morbidity among the Pakistani population (45, 46).

Pakistan urgently needs to develop and formulate a prevention and control strategy for obesity at the national level by implementing the World Health Organization (WHO) policies and recommendations for preventing obesity and diabetes in the Eastern Mediterranean Region (47). The government and non-government organizations should initiate and promote public awareness programs about the impact of a healthy diet and physical activity on individual and national health. School and college-based awareness and intervention pogroms should be initiated across the country for promoting healthy dietary habits and physical activity among the younger population. Adopting healthy dietary habits and sufficient physical activity could reduce the high BMI-related CVD burden in Pakistan (48).

Overall, male showed less improvement and high burden in CVD, IHD, and IS ASMR and age-standardized DALYs rate attributable to dietary risks and high BMI than the female population across China and Pakistan. Generally, men showed poor dietary habits and lower Healthy Eating Index (HEI) scores (49), a higher prevalence of obesity (50), and experienced earlier (10–15 years) coronary heart disease than women. Moreover, women had a lower incidence of CVD than men due to estrogen’s protective effect on the cardiovascular system (51, 52). Our findings are coinciding with several previous studies (7, 11, 53).

### Age-period-cohort effect on CVD burden attributable to dietary risks and high BMI

We found that the risk ratio of CVD mortality and DALYS showed a linear association with age. Age is an independent risk factor of CVD and the proportion of CVD incidence increased with aging among both men and women (54, 55). The age effect on CVD burden attributable to modifiable risk factors was remarkable in China. Moreover, the risk ratio of premature CVD mortality (<70 years) attributable to high BMI was higher among the Chinese population. Between 1993 and 2015, the prevalence of overweight (26.6 to 41.3%), obesity (4.2 to 15.7%), and abdominal obesity (20.2 to 46.9%) increased among Chinese adults aged 18–80 years. The absolute increase in BMI was positively correlated with age and older people showed a marked increasing trend in obesity (56) and a higher risk of CVD mortality and morbidity (20). On the other hand, the Pakistani population showed a higher risk ratio of premature CVD mortality attributable to dietary risk. Several factors such as malnutrition (28) poverty and low diet quality (29) poor dietary habits (30) and a poor healthcare system (57) may contribute to the increased risk of diet-related premature CVD mortality in Pakistan.

In our findings, the risk ratio of period effect on CVD burden attributable to modifiable risk factors significantly increased across both countries. The period effect was generally higher for CVD mortality and DALYs attributable to high BMI notably in Pakistan. Pakistan is facing twin problems of poor nutrition and over-nutrition and around 5.4 million school-aged children will be obese by 2030 (42). The increasing risk of BMI-related CVD burden in Pakistan could be attributed to the high trend of fast food, urbanization, moderation, and low physical activity. The fast food industry is the 2nd largest in Pakistan with 180 million consumers (58). The trend of fast food consumption is tremendously increasing among the young population (43). Due to rapid urbanization and modernization people have adopted a westernized high-fat diet over a traditional low-fat diet (43). Moreover, Pakistani adolescents had a low level of physical activity, and less than one-fifth achieved recommended levels (44).

We observed that compared to the reference cohort, the risk ratio of cohort effect on CVD burden attributable to dietary risks and high BMI showed a similar monotonic downward pattern among the Chinese and Pakistani populations. Early birth cohorts in China had a higher risk ratio of CVD mortality and DALYs. However, recent birth cohorts in China showed relatively a lower risk ratio of CVD mortality and DALYs than in Pakistan. Advancement in techniques for diagnosis, management, treatment, improvement in healthcare facilities, early-life nutrition, education level, and socioeconomic status may play a critical role in the lower risk ratio of CVD burden in the recent birth cohorts in China (17, 59, 60). In accordance with our findings, several studies observed that early birth cohorts showed higher risk and recent birth cohorts experienced a lower risk of CVD burden in China (17, 59, 61, 62).

Our study has several limitations. First, our findings are obtained from GBD data and all GBD limitations are apply to our results as mentioned previously (63). Second, we observed epidemiological trends of CVD mortality and morbidity attributable to overall dietary risks and could not find for the individual dietary risk factor. Third, the age-period-cohort effect on CVD burden attributable to dietary risks and high BMI was not conducted for different genders and at the regional level across China and Pakistan.

## Conclusion

Our findings indicate that the all-ages CVD deaths and DALYs due to dietary risks and high BMI significantly increased across China and Pakistan from 1990 to 2019. The diet-related CVD ASMR and the age-standardized DALYs rate significantly decreased in China. However, the ASMR of CVD and the age-standardized DALYs rate attributable to dietary risks significantly increased in Pakistan. Both countries showed a marked increasing trend of CVD ASMR and the age-standardized DALYs rate attributable to high BMI. Age-period-cohort analysis showed a higher risk ratio of premature CVD mortality (<70 years) among Chinese attributable to high BMI and Pakistani attributable to dietary risks. In China, early birth cohorts showed higher risk and recent birth cohorts experienced a lower risk of CVD burden compared with Pakistan. Besides government-level initiatives, proper nursing care could play a significant role in preventing and controlling premature CVD mortality across China and Pakistan (64). These findings can provide evidence-based references for health-policy makers to priorities strategies for the premature CVD burden attributable to dietary risk and high BMI across China and Pakistan.

## Data availability statement

The original contributions presented in the study are included in the article/Supplementary material, further inquiries can be directed to the corresponding authors.

## Ethics statement

Ethical approval was not provided for this study on human participants because ethical review and approval was not required for the study on human participants in accordance with the local legislation and institutional requirements. The requirement for informed consent was waived. The ethics committee waived the requirement of written informed consent for participation.

## Author contributions

XY, WY, and Nawsherwan: study design and critical revision of manuscript and writing— review and editing. XY, SM, and Nawsherwan: data collection and analysis and drafting the manuscript. Nawsherwan and SM: data collection. Nawsherwan and SM: data analysis. WY and Nawsherwan: writing—original draft preparation. Nawsherwan: visualization. XY and WY: supervision. WY: project administration. All authors have read and agreed to the published version of the manuscript.

## Conflict of interest

The authors declare that the research was conducted in the absence of any commercial or financial relationships that could be construed as a potential conflict of interest.

## Publisher’s note

All claims expressed in this article are solely those of the authors and do not necessarily represent those of their affiliated organizations, or those of the publisher, the editors and the reviewers. Any product that may be evaluated in this article, or claim that may be made by its manufacturer, is not guaranteed or endorsed by the publisher.

## Footnotes

^1^ http://ghdx.healthdata.org/gbd-results-tool
