# Supplementary material for: Epidemiological trend and age-period-cohort effects on cardiovascular disease mortality and disability-adjusted life years attributable to dietary risks and high body mass index at the regional and country level across China and Pakistan
Source: Front Nutr. 2023 Jun 6;10:1158769. doi: 10.3389/fnut.2023.1158769 (PMC10280070; doi:10.3389/fnut.2023.1158769)
Supplement: Supplementary file 1 [file Data_Sheet_1.docx]

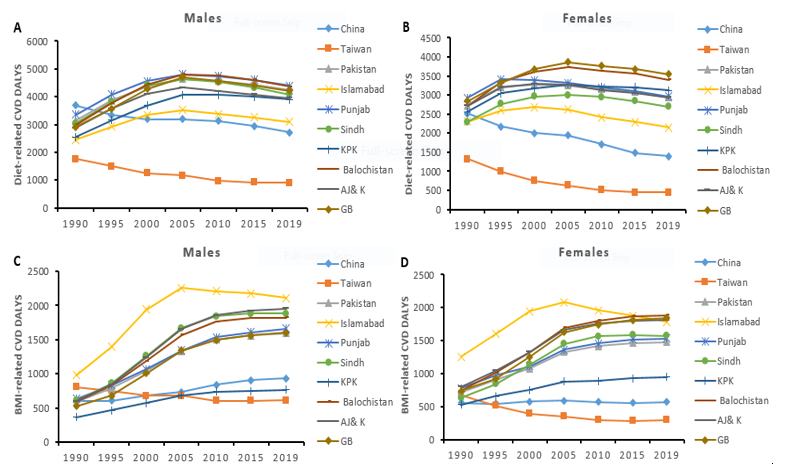


**Fig S1:** Temporal trend of CVD age-standardized DALYS rate (per 100,000) attributable to dietary risks (A and B) and high BMI (C and D) in males and females across China and Pakistan.


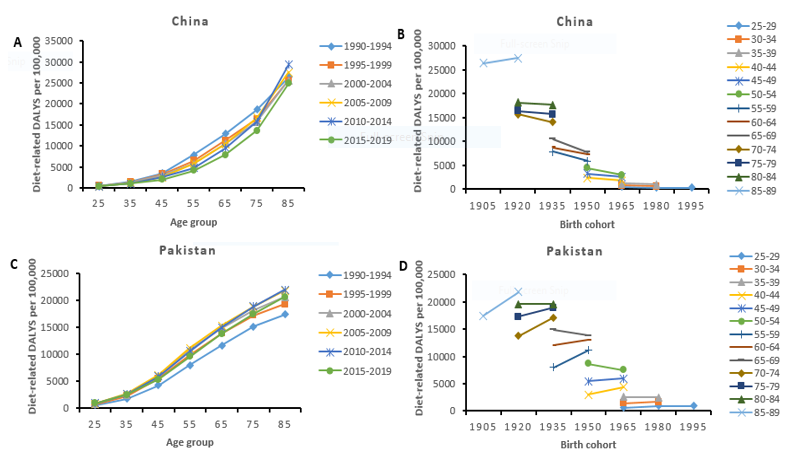


**Figure S2:** Diet-related age-specific CVD DALYS rate by period (A and C) and cohort-specific CVD DALYS rate by age group (B and D) across China and Pakistan from 1990 to 2019.


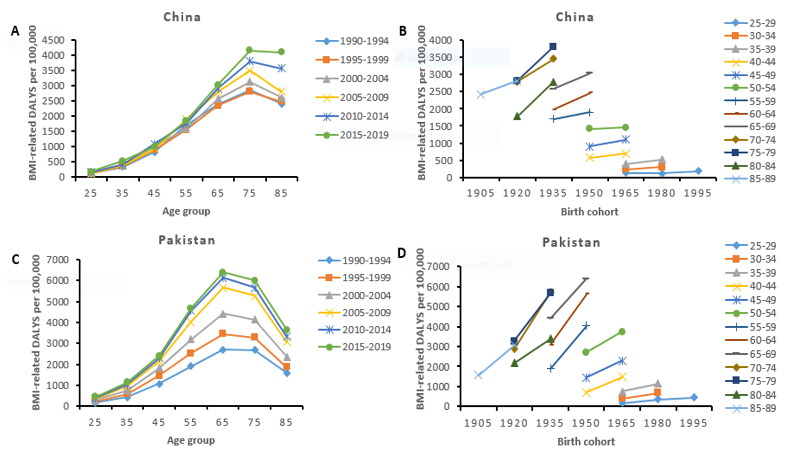


**Figure S3:** High BMI-related age-specific CVD DALYS rate by period (A and C) and cohort-specific CVD DALYS rate by age group (B and D) across China and Pakistan from 1990 to 2019.
