# Supplementary material for: Epidemiological trend and age-period-cohort effects on cardiovascular disease mortality and disability-adjusted life years attributable to dietary risks and high body mass index at the regional and country level across China and Pakistan
Source: Front Nutr. 2023 Jun 6;10:1158769. doi: 10.3389/fnut.2023.1158769 (PMC10280070; doi:10.3389/fnut.2023.1158769)
Supplement: Supplementary file 2 [file Data_Sheet_2.docx]

**Table S1:** The temporal trend in the burden of IHD mortality attributable to the dietary risks and high BMI for both sexes across China and Pakistan from 1990 to 2019.

| Dietary risks | ASMR/100,000 | | | Deaths, n×10,000 | | |
| --- | --- | --- | --- | --- | --- | --- |
| IHD | 1990 (95%UI) | 2019 (95%UI) | AAPC (95%CI) | 1990 (95%UI) | 2019 (95%UI) | AAPC (95%CI) |
| China | 58 (70, 47) | 60 (74, 46) | 0.1 (-0.2, 0.2) | 38 (44, 31) | 99 (122, 76) | 3.4 (3.1, 3.6) |
| Taiwan | 35 (43, 26) | 17 (23, 12) | -2.4 (-2.8, -2.0) | 0.4 (0.5, 0.3) | 0.6 (0.9, 0.4) | 1.7 (1.3, 2.2) |
| Pakistan | 91 (109, 73) | 118 (147, 93) | 0.9 (0.8, 1.0) | 4.8 (5.8, 3.9) | 12 (15, 9.3) | 3.2 (3.0, 3.3) |
| Islamabad | 80 (103, 59) | 98 (126, 72) | 0.8 (0.6, 0.9) | 0.01 (0.02, 0.01) | 0.06 (0.09, 0.04) | 5.0 (4.9, 5.2) |
| Punjab | 97 (119, 76) | 121 (153, 95) | 0.8 (0.7, 0.8) | 3.1 (3.8, 2.4) | 6.9 (8.8, 5.4) | 2.9 (2.7, 3.0) |
| Sindh | 88 (113, 65) | 118 (155, 90) | 1.0 (1.0, 1.1) | 0.8 (1.1, 0.6) | 2.4 (3.2, 1.8) | 3.7 (3.6, 3.8) |
| KPK | 73 (97, 52) | 107 (143, 77) | 1.3 (1.2, 1.4) | 0.5 (0.7, 0.3) | 1.6 (2.1, 1.1) | 3.7 (3.6, 3.9) |
| Balochistan | 88 (116, 61) | 125 (164, 91) | 1.2 (1.1, 1.4) | 0.2 (0.2, 0.1) | 0.5 (0.6, 0.3) | 3.3 (3.2, 3.4) |
| AJ& K | 86 (113, 65) | 118 (150, 87) | 1.1 (1.0, 1.2) | 0.1 (0.1, 0.08) | 0.2 (0.3, 0.1) | 2.9 (2.8, 3.0) |
| GB | 83 (114, 59) | 120 (165, 83) | 1.3 (1.1, 1.5) | 0.02 (0.03, 0.01) | 0.08 (0.1, 0.05) | 4.6 (4.4, 4.8) |
| High BMI |  |  |  |  |  |  |
| China | 5.1 (12, 1.1) | 11 (20, 4.5) | 2.7 (2.5, 3.0) | 3.9 (8.9, 0.9) | 20 (36, 8.4) | 5.8 (5.6, 6.0) |
| Taiwan | 8.2 (14, 3.5) | 5.5 (9.3, 2.6) | -1.4 (-1.7, -1.1) | 0.1 (0.1, 0.05) | 0.2 (0.3, 0.1) | 2.1 (1.7, 2.6) |
| Pakistan | 10 (22, 2.6) | 29 (46, 15) | 3.7 (3.5, 3.9) | 0.5 (1.2, 0.1) | 3.3 (5.4, 1.7) | 6.3 (6.1, 6.4) |
| Islamabad | 18 (31, 8.4) | 43 (65, 26) | 3.1 (2.9, 3.3) | 0.01 (0.01, 0.002) | 0.03 (0.05, 0.02) | 7.6 (7.3, 7.8) |
| Punjab | 11 (23, 2.8) | 29 (48, 15) | 3.5 (3.4, 3.7) | 0.3 (0.8, 0.09) | 1.9 (3.1, 1.0) | 5.9 (5.8, 6.1) |
| Sindh | 10 (23, 2.8) | 35 (57, 19) | 4.3 (4.2, 4.4) | 0.1 (0.2, 0.03) | 0.8 (1.3, 0.4) | 7.2 (7.1, 7.3) |
| KPK | 5.9 (15, 1.1) | 13 (29, 3.8) | 2.9 (2.7, 3.2) | 0.05 (0.1, 0.009) | 0.2 (0.4, 0.06) | 5.5 (5.3, 5.6) |
| Balochistan | 10 (23, 2.5) | 33 (56, 17) | 4.2 (4.0, 4.4) | 0.02 (0.05, 0.006) | 0.1 (0.2, 0.08) | 6.5 (6.4, 6.7) |
| AJ& K | 10 (22, 2.8) | 36 (57, 20) | 4.4 (4.3, 4.5) | 0.01 (0.03, 0.003) | 0.08 (0.1, 0.05) | 6.5 (6.4, 6.6) |
| GB | 8.9 (21, 2.1) | 29 (50, 13) | 4.2 (4.0, 4.4) | 0.01 (0.01, 0.001) | 0.02 (0.04, 0.01) | 7.7 (7.5, 7.9) |

**Abbreviations:** KPK, Khyber Pakhtunkhwa; AJ& K, Azad Jammu & Kashmir; GB, Gilgit Baltistan; IHD, ischemic heart disease; ASMR, age-standardized mortality rate; BMI, body mass index; AAPC, average annual percent change.

**Table S2:** The temporal trend in the burden of IHD DALYs attributable to the dietary risk and high BMI for both sexes across China and Pakistan from 1990 to 2019.

| Dietary risks | Age-standardized DALYs /100,000 | | | DALYs, n×10,000 | | |
| --- | --- | --- | --- | --- | --- | --- |
| IHD | 1990 (95%UI) | 2019 (95%UI) | AAPC (95%CI) | 1990 (95%UI) | 2019 (95%UI) | AAPC (95%CI) |
| China | 1176 (1375, 965) | 1060 (1288, 832) | -0.4 (-0.5, -0.2) | 977  (1148, 799) | 2004  (2445, 1560) | 2.5 (2.3, 2.7) |
| Taiwan | 640 (784, 489) | 341 (457, 241) | -2.1 (-2.4, -1.9) | 9.6 (11, 7.3) | 13 (17, 9.2) | 1.1 (0.7, 1.5) |
| Pakistan | 2112 (2512, 1702) | 2766 (3485, 2179) | 1.0 (0.9, 1.0) | 127 (150,103) | 348 (439, 273) | 3.6 (3.4, 3.7) |
| Islamabad | 1718 (2269, 1227) | 2064 (2805, 1472) | 0.7 (0.5, 0.8) | 0.4 (0.5, 0.3) | 1.9 (2.8, 1.3) | 5.3 (5.1, 5.4) |
| Punjab | 2266 (2757, 1805) | 2845 (3606, 2220) | 0.8 (0.7, 0.9) | 79 (96, 63) | 201 (256, 155) | 3.3 (3.2, 3.4) |
| Sindh | 2021 (2584, 1491) | 2738 (3649, 2052) | 1.1 (1.0, 1.2) | 23 (30, 17) | 73 (99, 54) | 4.0 (3.9, 4.1) |
| KPK | 1689 (2231, 1214) | 2520 (3428, 1802) | 1.4 (1.3, 1.5) | 14 (19, 10) | 46 (63, 32) | 4.0 (3.8, 4.2) |
| Balochistan | 2046 (2768, 1385) | 2963 (3985, 2126) | 1.3 (1.2, 1.4) | 5.4 (7.4, 3.5) | 15 (21, 10) | 3.8 (3.6, 3.9) |
| AJ& K | 1973 (2605, 1488) | 2623 (3460, 1902) | 1.0 (0.9, 1.1) | 2.7 (3.6, 2.1) | 6.8 (9.2, 4.9) | 3.2 (3.1, 3.4) |
| GB | 1968 (2690, 1367) | 2871 (3983, 1966) | 1.4 (1.1, 1.6) | 0.6 (0.9, 0.4) | 2.6 (3.7, 1.8) | 4.8 (4.5, 5.0) |
| High BMI |  |  |  |  |  |  |
| China | 127 (286, 30) | 257 (447, 112) | 2.5 (2.3, 2.6) | 118 (264, 28) | 507 (874, 223) | 5.2 (5.0, 5.3) |
| Taiwan | 183 (311, 80) | 143 (231, 71) | -0.9 (-1.2, -0.5) | 3.1 (5.1, 1.3) | 5.3 (8.7, 2.6) | 2.0 (1.7, 2.4) |
| Pakistan | 276 (594, 74) | 803 (1290, 417) | 3.8 (3.6, 4.0) | 17 (37, 4.7) | 109 (178, 57) | 6.5 (6.4, 6.7) |
| Islamabad | 472 (816, 221) | 1075 (1610, 662) | 3.0 (2.7, 3.2) | 0.1 (0.2, 0.06) | 1.1 (1.7, 0.6) | 7.7 (7.5, 7.9) |
| Punjab | 302 (649, 79) | 831 (1314, 425) | 3.6 (3.4, 3.8) | 11 (23, 2.9) | 63 (100, 32) | 6.3 (6.1, 6.5) |
| Sindh | 285 (602, 76) | 972 (1549, 541) | 4.4 (4.2, 4.6) | 3.6 (7.6, 0.9) | 28 (46, 15) | 7.4 (7.3, 7.6) |
| KPK | 163 (402, 29) | 378 (810, 108) | 3.0 (2.8, 3.2) | 1.5 (3.7, 0.2) | 7.5 (16, 2.1) | 5.6 (5.4, 5.9) |
| Balochistan | 278 (629, 69) | 931 (1566, 470) | 4.3 (4.0, 4.7) | 0.7 (1.8, 0.2) | 5.4 (9.2, 2.6) | 6.9 (6.7, 7.1) |
| AJ& K | 285 (610, 80) | 974 (1543, 551) | 4.4 (4.2, 4.6) | 0.4 (0.8, 0.1) | 2.7 (4.4, 1.5) | 6.8 (6.6, 6.9) |
| GB | 246 (576, 56) | 828 (1420, 385) | 4.3 (4.1, 4.5) | 0.1 (0.2, 0.02) | 0.8 (1.4, 0.3) | 7.9 (7.7, 8.0) |

**Abbreviations:** KPK, Khyber Pakhtunkhwa; AJ& K, Azad Jammu & Kashmir; GB, Gilgit Baltistan; IHD, ischemic heart disease; DALYs, disability-adjusted life years; BMI, body mass index; AAPC, average annual percent change.

**Table S3:** The temporal trend in the burden of IHD ASMR and DALYs attributable to the dietary risk and high BMI in males and females across China and Pakistan from 1990 to 2019.

| IHD ASMR | Male (AAPC (95%CI) | | Female (AAPC (95%CI) | |
| --- | --- | --- | --- | --- |
|  | Dietary risks | High BMI | Dietary risks | High BMI |
| China | 0.4 (0.1, 0.6) | 3.1 (2.7, 3.5) | -0.3 (-0.5, -0.1) | 2.2 (2.0, 2.4) |
| Taiwan | -1.9 (-2.2, -1.5) | -0.5 (-0.9, -0.1) | -3.0 (-3.4, -2.6) | -2.6 (-3.1, -2.1) |
| Pakistan | 1.3 (1.2, 1.4) | 4.2 (4.1, 4.4) | 0.4 (0.4, 0.5) | 3.0 (2.9, 3.2) |
| Islamabad | 1.2 (1.1, 1.3) | 3.8 (3.5, 4.1) | 0.1 (-0.1, 0.2) | 2.1 (1.9, 2.3) |
| Punjab | 1.2 (1.1, 1.3) | 4.1 (3.8, 4.4) | 0.2 (0.1, 0.3) | 2.9 (2.7, 3.1) |
| Sindh | 1.3 (1.2, 1.4) | 4.7 (4.5, 4.9) | 0.8 (0.7, 0.8) | 3.7 (3.6, 3.9) |
| KPK | 1.7 (1.6, 1.8) | 3.2 (3.0, 3.4) | 0.9 (0.8, 1.0) | 2.5 (2.4, 2.7) |
| Balochistan | 1.5 (1.4, 1.7) | 4.6 (4.4, 4.9) | 0.9 (0.8, 1.0) | 3.7 (3.5, 3.9) |
| AJ& K | 1.5 (1.4, 1.6) | 5.1 (4.9, 5.3) | 0.7 (0.6, 0.8) | 3.6 (3.4, 3.8) |
| GB | 1.5 (1.2, 1.8) | 4.6 (4.5, 4.8) | 1.0 (0.8, 1.2) | 3.8 (3.6, 4.0) |
| IHD DALYs |  |  |  |  |
| China | -0.1 (-0.2, 0.2) | 3.0 (2.7, 3.3) | -0.9 (-1.0, -0.7) | 1.7 (1.5, 1.8) |
| Taiwan | -1.5 (-1.9, -1.0) | 0.1 (-0.4, 0.5) | -3.1 (-3.4, -2.7) | -2.3 (-2.7, -1.9) |
| Pakistan | 1.4 (1.3, 1.5) | 4.3 (4.1, 4.5) | 0.4 (0.3, 0.5) | 3.1 (2.8, 3.4) |
| Islamabad | 1.1 (1.0, 1.3) | 3.6 (3.3, 4.0) | -0.1 (-0.2, 0.1) | 1.9 (1.8, 2.1) |
| Punjab | 1.3 (1.2, 1.4) | 4.2 (4.0, 4.4) | 0.2 (0.1, 0.3) | 2.8 (2.4, 3.2) |
| Sindh | 1.4 (1.2, 1.5) | 4.8 (4.5, 5.0) | 0.7 (0.6, 0.8) | 3.8 (3.6, 4.0) |
| KPK | 1.8 (1.6, 2.0) | 3.3 (3.1, 3.6) | 0.8 (0.7, 1.0) | 2.5 (2.3, 2.7) |
| Balochistan | 1.6 (1.5, 1.8) | 4.7 (4.5, 5.0) | 0.9 (0.8, 1.0) | 3.7 (3.5, 3.9) |
| AJ& K | 1.5 (1.3, 1.7) | 5.0 (4.8, 5.3) | 0.5 (0.4, 0.6) | 3.6 (3.3, 3.9) |
| GB | 1.6 (1.4, 1.8) | 4.8 (4.6, 4.9) | 1.0 (0.8, 1.2) | 3.8 (3.6, 4.0) |

**Abbreviations:** KPK, Khyber Pakhtunkhwa; AJ& K, Azad Jammu & Kashmir; GB, Gilgit Baltistan; IHD, ischemic heart disease; ASMR, age-standardized mortality rate; DALYS, disability-adjusted life years; BMI, body mass index; AAPC, average annual percent change.

**Table S4:** The temporal trend in the burden of IS mortality attributable to the dietary risks and high BMI for both sexes across China and Pakistan from 1990 to 2019.

| Dietary risks | ASMR/100,000 | | | Deaths, n×1000 | | |
| --- | --- | --- | --- | --- | --- | --- |
| IS | 1990 (95%UI) | 2019 (95%UI) | AAPC (95%CI) | 1990 (95%UI) | 2019 (95%UI) | AAPC (95%CI) |
| China | 18 (26, 12) | 15 (22, 10) | -0.5 (-0.9, -0.2) | 125 (175, 84) | 283 (404, 179) | 2.8 (2.6, 3.1) |
| Taiwan | 13 (19, 8.5) | 3.9 (5.9, 2.3) | -4.0 (-4.4, -3.6) | 1.6 (2.3, 1.1) | 1.6 (2.4, 0.9) | -0.1 (-0.4, 0.2) |
| Pakistan | 11 (18, 7.1) | 13 (19, 8.6) | 0.4 (0.4, 0.5) | 5.4 (8.8, 3.3) | 10 (15, 6.8) | 2.3 (2.2, 2.3) |
| Islamabad | 12 (18, 7.4) | 13 (19, 7.9) | 0.2 (0.1, 0.3) | 0.02 (0.03, 0.01) | 0.07 (0.1, 0.04) | 4.3 (4.2, 4.4) |
| Punjab | 12 (19,7.5) | 13 (19, 8.7) | 0.3 (0.2, 0.3) | 3.6 (5.7, 2.2) | 6.2 (9.2, 4.1) | 1.9 (1.9, 2.0) |
| Sindh | 9.6 (16, 5.4) | 11 (16, 6.7) | 0.5 (0.4, 0.5) | 0.8 (1.3, 0.4) | 1.7 (2.6, 1.1) | 2.8 (2.7, 2.8) |
| KPK | 10 (19, 6.1) | 14 (22, 8.9) | 1.0 (0.9, 1.1) | 0.7 (1.2, 0.3) | 1.7 (2.8, 1.1) | 3.2 (3.0, 3.3) |
| Balochistan | 9.9 (16, 5.6) | 12 (19, 7.5) | 0.8 (0.7, 0.9) | 0.1 (0.3, 0.1) | 0.3 (0.5, 0.2) | 2.4 (2.3, 2.5) |
| AJ& K | 11 (18, 6.7) | 14 (20, 8.6) | 0.7 (0.6, 0.8) | 0.1 (0.2, 0.07) | 0.2 (0.3, 0.1) | 2.2 (2.1, 2.3) |
| GB | 10 (19, 5.6) | 13 (23, 7.7) | 0.9 (0.7, 1.1) | 0.02 (0.04, 0.01) | 0.07 (0.1,0.04) | 4.2 (4.0, 4.5) |
| High BMI |  |  |  |  |  |  |
| China | 2.5 (5.6, 0.5) | 4.4 (8.1, 1.8) | 1.9 (1.6, 2.3) | 20 (44, 4.8) | 87 (154, 37) | 5.1 (4.8, 5.4) |
| Taiwan | 3.7 (6.4, 1.5) | 1.2 (2.3, 0.5) | -3.5 (-4.0, -3.1) | 0.5 (0.9, 0.2) | 0.5 (0.9, 0.2) | -0.2 (-0.7, 0.2) |
| Pakistan | 2.1 (4.8, 0.4) | 5.1 (8.5, 2.5) | 3.1 (3.0, 3.2) | 1.1 (2.5, 0.2) | 5.1 (8.3, 2.5) | 5.3 (5.1, 5.5) |
| Islamabad | 4.8 (8.7, 2.1) | 9.3 (14, 5.2) | 2.3 (2.2, 2.5) | 0.01 (0.01, 0.004) | 0.06 (0.09, 0.03) | 6.6 (6.4, 6.8) |
| Punjab | 2.3 (5.3, 0.5) | 5.4 (9.1, 2.5) | 3.0 (2.8, 3.3) | 0.7 (1.7, 0.2) | 3.1 (5.1, 1.5) | 5.0 (4.9, 5.1) |
| Sindh | 1.9 (4.5, 0.4) | 5.3 (9.1, 2.7) | 3.6 (3.4, 3.8) | 0.2 (0.4, 0.04) | 1.1 (1.7, 0.5) | 6.3 (6.2, 6.4) |
| KPK | 1.3 (3.5, 0.2) | 2.8 (6.1, 0.7) | 2.5 (2.4, 2.7) | 0.1 (0.2, 0.01) | 0.4 (0.8, 0.1) | 4.9 (4.6, 5.1) |
| Balochistan | 1.9 (4.4, 0.4) | 5.3 (9.2, 2.5) | 3.7 (3.5, 3.9) | 0.04 (0.1, 0.009) | 0.2 (0.3, 0.09) | 5.6 (5.4, 5.8) |
| AJ& K | 2.2 (5.2, 0.5) | 6.8 (11, 3.5) | 3.9 (3.7, 4.1) | 0.02 (0.06, 0.007) | 0.2 (0.2, 0.08) | 5.8 (5.7, 6.0) |
| GB | 1.7 (4.6, 0.3) | 5.1 (10, 2.1) | 3.7 (3.4, 4.0) | 0.01 (0.01, 0.001) | 0.03 (0.06, 0.01) | 7.1 (6.9, 7.3) |

**Abbreviations:** KPK, Khyber Pakhtunkhwa; AJ& K, Azad Jammu & Kashmir; GB, Gilgit Baltistan; IS, ischemic stroke; ASMR, age-standardized mortality rate; BMI, body mass index; AAPC, average annual percent change.

**Table S5:** The temporal trend in the burden of IS DALYs attributable to the dietary risk and high BMI for both sexes across China and Pakistan from 1990 to 2019.

| Dietary risks | Age-standardized DALYs /100,000 | | | DALYs, n×1000 | | |
| --- | --- | --- | --- | --- | --- | --- |
| IS | 1990 (95%UI) | 2019 (95%UI) | AAPC (95%CI) | 1990 (95%UI) | 2019 (95%UI) | AAPC (95%CI) |
| China | 412 (557, 284) | 357 (496, 239) | -0.5 (-0.7, -0.2) | 3399 (4518, 2374) | 7082 (9757,4722) | 2.6 (2.4, 2.8) |
| Taiwan | 285 (392, 188) | 111 (156, 73) | -3.2 (-3.5, -3.0) | 42 (57, 28) | 42 (60, 27) | 0.1 (-0.2, 0.2) |
| Pakistan | 221 (348, 141) | 256 (366, 170) | 0.5 (0.5, 0.6) | 121 (189, 77) | 266 (378, 180) | 2.8 (2.7, 2.8) |
| Islamabad | 219 (329, 135) | 239 (356, 148) | 0.3 (0.2, 0.4) | 0.4 (07, 0.3) | 1.8 (2.7, 1.2) | 4.8 (4.7, 4.9) |
| Punjab | 237 (372, 153) | 265 (385, 173) | 0.4 (0.3, 0.4) | 77 (121, 50) | 156 (221, 103) | 2.5 (2.4, 2.6) |
| Sindh | 189 (309, 113) | 220 (321, 142) | 0.5 (0.5, 0.6) | 19 (31, 11) | 48 (68, 31) | 3.2 (3.1, 3.3) |
| KPK | 206 (366, 126) | 271 (431, 173) | 0.9 (0.9, 1.0) | 16 (28, 9.8) | 41 (65, 26) | 3.3 (3.2, 3.5) |
| Balochistan | 192 (317, 113) | 243 (368, 154) | 0.8 (0.7, 0.9) | 4.4 (7.3, 2.6) | 10 (14, 6.4) | 2.9 (2.8, 3.0) |
| AJ& K | 212 (355, 132) | 259 (385, 165) | 0.7 (0.6, 0.8) | 2.7 (4.6, 1.7) | 5.8 (8.7, 3.7) | 2.7 (2.6, 2.8) |
| GB | 198 (365, 117) | 257 (441, 154) | 0.9 (0.7, 1.2) | 0.5 (1.1, 0.3) | 1.9 (3.1, 1.1) | 4.3 (4.1, 4.4) |
| High BMI |  |  |  |  |  |  |
| China | 71 (154, 17) | 131 (223, 59) | 2.2 (1.9, 2.4) | 647 (1391, 161) | 2722 (4594,1234) | 5.1 (4.9, 5.3) |
| Taiwan | 104 (172, 46) | 59 (94, 30) | -2.0 (-2.2, -1.7) | 17 (28, 7.9) | 22 (35, 11) | 0.8 (0.5, 1.0) |
| Pakistan | 53 (117, 13) | 131 (211, 69) | 3.2 (3.0, 3.3) | 32 (69, 8.1) | 155 (249, 83) | 5.7 (5.5, 5.8) |
| Islamabad | 117 (196, 55) | 225 (336, 138) | 2.4 (2.2, 2.6) | 0.3 (0.4, 0.2) | 2.1 (2.9, 1.3) | 7.0 (6.8, 7.2) |
| Punjab | 58 (130, 14) | 139 (223, 73) | 3.1 (2.8, 3.3) | 20 (45, 5.1) | 93 (148, 49) | 5.4 (5.2, 5.6) |
| Sindh | 50 (114, 13) | 142 (225, 79) | 3.6 (3.5, 3.8) | 5.7 (12, 1.5) | 36 (54, 20) | 6.5 (6.5, 6.6) |
| KPK | 36 (88, 7) | 73 (143, 21) | 2.5 (2.3, 2.6) | 3.1 (7.6, 0.5) | 13 (24, 3.7) | 4.9 (4.7, 5.1) |
| Balochistan | 48 (111, 12) | 138 (229, 70) | 3.7 (3.5, 3.8) | 1.2 (2.7, 0.3) | 6.7 (11, 3.5) | 6.0 (5.8, 6.1) |
| AJ& K | 57 (124, 16) | 171 (268, 96) | 3.9 (3.7, 4.1) | 0.7 (1.7, 0.2) | 4.4 (6.8, 2.5) | 6.1 (6.0, 6.2) |
| GB | 46 (114, 10) | 131 (239, 60) | 3.7 (3.5, 3.9) | 0.2 (0.3, 0.03) | 1.2 (2.1, 0.5) | 7.1 (6.9, 7.3) |

**Abbreviations:** KPK, Khyber Pakhtunkhwa; AJ& K, Azad Jammu & Kashmir; GB, Gilgit Baltistan; IS, ischemic stroke; DALYs, disability-adjusted life years; BMI, body mass index; AAPC, average annual percent change.

**Table S6:** The temporal trend in the burden of IS ASMR and DALYs attributable to the dietary risk and high BMI in males and females across China and Pakistan from 1990 to 2019.

| IS ASMR | Male (AAPC (95%CI) | | Female (AAPC (95%CI) | |
| --- | --- | --- | --- | --- |
|  | Dietary risks | High BMI | Dietary risks | High BMI |
| China | -0.1 (-0.6, 0.4) | 2.4 (1.9, 3.0) | -1.1 (-1.6, -0.7) | 1.3 (1.0, 1.6) |
| Taiwan | -3.6 (-3.9, -3.2) | -2.5 (-3.0, -2.1) | -4.5 (-4.9, -4.1) | -4.7 (-5.2, -4.1) |
| Pakistan | 0.3 (0.2, 0.3) | 3.2 (3.0, 3.3) | 0.6 (0.6, 0.7) | 3.0 (2.8, 3.2) |
| Islamabad | 0.1 (-0.1, 0.3) | 2.7 (2.4, 2.9) | 0.2 (0.1, 0.3) | 2.0 (1.8, 2.1) |
| Punjab | 0.2 (0.1, 0.3) | 3.0 (2.9, 3.1) | 0.4 (0.3, 0.5) | 2.8 (2.7, 3.0) |
| Sindh | 0.2 (0.1, 0.3) | 3.5 (3.4, 3.7) | 0.9 (0.8, 1.0) | 3.7 (3.6, 3.8) |
| KPK | 0.7 (0.6, 0.8) | 2.2 (2.0, 2.3) | 1.1 (0.9, 1.2) | 2.5 (2.2, 2.8) |
| Balochistan | 0.5 (0.4, 0.6) | 3.6 (3.4, 3.9) | 1.1 (1.0, 1.2) | 3.7 (3.6, 3.9) |
| AJ& K | 0.5 (0.4, 0.5) | 4.1 (3.9, 4.2) | 0.9 (0.8, 1.0) | 3.6 (3.5, 3.8) |
| GB | 0.6 (0.4, 0.7) | 3.6 (3.4, 3.7) | 1.2 (1.0, 1.4) | 3.8 (3.6, 3.9) |
| IS DALYs |  |  |  |  |
| China | -0.1 (-0.6, 0.3) | 2.6 (2.1, 3.2) | -0.9 (-1.2, -0.6) | 1.7 (1.5, 1.9) |
| Taiwan | -3.0 (-3.3, -2.8) | -1.6 (-1.8, -1.3) | -3.3 (-3.6, -3.0) | -2.2 (-2.4, -1.9) |
| Pakistan | 0.4 (0.3, 0.5) | 3.3 (3.1, 3.4) | 0.6 (0.5, 0.7) | 3.0 (2.9, 3.1) |
| Islamabad | 0.3 (0.2, 0.4) | 2.6 (2.4, 2.9) | 0.2 (0.1, 0.3) | 2.0 (1.8, 2.1) |
| Punjab | 0.4 (0.3, 0.4) | 3.1 (3.0, 3.3) | 0.4 (0.3, 0.5) | 2.9 (2.8, 3.0) |
| Sindh | 0.4 (0.3, 0.5) | 3.6 (3.5, 3.8) | 0.8 (0.8, 0.9) | 3.6 (3.6, 3.7) |
| KPK | 0.8 (0.7, 0.9) | 2.2 (2.0, 2.3) | 1.0 (0.9, 1.1) | 2.5 (2.2, 2.7) |
| Balochistan | 0.7 (0.6, 0.7) | 3.7 (3.5, 4.0) | 1.1 (1.0, 1.2) | 3.7 (3.5, 3.9) |
| AJ& K | 0.6 (0.5, 0.7) | 4.1 (3.9, 4.3) | 0.8 (0.7, 0.9) | 3.6 (3.5, 3.7) |
| GB | 0.7 (0.4, 0.9) | 3.7 (3.5, 3.8) | 1.1 (0.9, 1.3) | 3.7 (3.6, 3.8) |

**Abbreviations:** KPK, Khyber Pakhtunkhwa; AJ& K, Azad Jammu & Kashmir; GB, Gilgit Baltistan; IS, ischemic stroke; ASMR, age-standardized mortality rate; DALYs, disability-adjusted life years; BMI, body mass index; AAPC, average annual percent change.
